# *Tet1* Deficiency Leads to Premature Ovarian Failure

**DOI:** 10.3389/fcell.2021.644135

**Published:** 2021-03-23

**Authors:** Linlin Liu, Huasong Wang, Guo_Liang Xu, Lin Liu

**Affiliations:** ^1^Department of Cell Biology and Genetics, College of Life Sciences, Nankai University, Tianjin, China; ^2^State Key Laboratory of Medicinal Chemical Biology, Nankai University, Tianjin, China; ^3^State Key Laboratory of Molecular Biology, Shanghai Institute of Biochemistry and Cell Biology, Chinese Academy of Sciences, Shanghai, China; ^4^Key Laboratory of Medical Epigenetics and Metabolism, Institutes of Biomedical Sciences, Medical College of Fudan University, Shanghai, China

**Keywords:** *Tet1*, aging, epigenetics, oocyte, premature ovarian failure

## Abstract

Tet enzymes participate in DNA demethylation and play critical roles in stem cell pluripotency and differentiation. DNA methylation alters with age. We find that *Tet1* deficiency reduces fertility and leads to accelerated reproductive failure with age. Noticeably, *Tet1*-deficient mice at young age exhibit dramatically reduced follicle reserve and the follicle reserve further decreases with age, phenomenon consistent with premature ovarian failure (POF) syndrome. Consequently, *Tet1-*deficient mice become infertile by reproductive middle age, while age matched wild-type mice still robustly reproduce. Moreover, by single cell transcriptome analysis of oocytes, *Tet1* deficiency elevates organelle fission, associated with defects in ubiquitination and declined autophagy, and also upregulates signaling pathways for Alzheimer’s diseases, but down-regulates X-chromosome linked genes, such as *Fmr1*, which is known to be implicated in POF. Additionally, *Line1* is aberrantly upregulated and endogenous retroviruses also are altered in *Tet1-*deficient oocytes. These molecular changes are consistent with oocyte senescence and follicle atresia and depletion found in premature ovarian failure or insufficiency. Our data suggest that *Tet1* enzyme plays roles in maintaining oocyte quality as well as oocyte number and follicle reserve and its deficiency can lead to POF.

## Introduction

Ten-eleven translocation (Tet) methylcytosine dioxygenases play a major role in shaping DNA methylation patterns through demethylation (Wu and Zhang, 2017). Tet enzymes have been demonstrated to play important roles in ESC pluripotency and differentiation (Ito et al., 2010; Koh et al., 2011). However, *Tet1* or *Tet2* -null mice are viable and overtly normal double *Tet1*/*Tet2*-deficient mice are also obtained (Dawlaty et al., 2011, 2013; Ko et al., 2011), but, some *Tet1*-deficient mice display a smaller body size at birth, which might reflect a developmental delay. Moreover, Tet1-mediated 5 hmC signals play important role in DNA demethylation during primordial germ cell (PGC) development and meiosis (Yamaguchi et al., 2012, 2013a; Hill et al., 2018). *Tet1* deficiency results in meiotic defects of PGCs, including impaired homologous pairing and recombination in meiotic germ cells (meiocytes) during fetal development, probably due to insufficient demethylation and failed activation of meiotic genes (Yamaguchi et al., 2013a), such that *Tet1-*deficient mice at young age already exhibit reduced number of oocytes in the ovary and subfertility (Yamaguchi et al., 2012, 2013a). It also will be interesting to investigate whether *Tet1* deficiency impacts reproductive aging of adult mice.

From the onset of reproductive maturity, organismal aging is characterized by declined fecundity, increased tissue dysfunction, and susceptibility to disease or mortality (Rando and Chang, 2012). The aging process is associated with altered epigenetic regulation, including DNA methylation, histone modification and chromatin remodeling (Zhang et al., 2020). DNA methylation involves in many aspects of cellular and molecular changes in aging (Lopez-Otin et al., 2013). Moreover, change to DNA methylation in aging has been used to predict the chronological age of human somatic tissues and individuals (Hannum et al., 2013; Horvath, 2013). Predictions have been made from heterogeneous samples such as lung, liver, and brain tissues as well as whole blood and peripheral blood mononuclear cells, and isolated CD4 + T cells, monocytes, and B cells (Horvath, 2013). Epigenetic clock sites have been defined also in multiple tissues in mouse (Stubbs et al., 2017) and age-related methylation changes between mouse and human were partially conserved (Maegawa et al., 2010). Deeper understanding of epigenetic alterations in aging and the molecular basis of the DNA methylation clock is of particular interest as epigenetic modifications can be reversed, allowing manipulation to potentially reverse aging and thus therapeutic potential (Rando and Chang, 2012; Cole et al., 2017; Chen and Kerr, 2019; Zhang et al., 2020). Nevertheless, it remains elusive whether *Tet* deficiency can impact aging in the adult.

Ovarian aging is mainly characterized by a sharp decrease in the number of oocyte and follicles and a decrease in oocyte quality (van Rooij et al., 2005; Broekmans et al., 2007, 2009; Tatone et al., 2008). The number of oocytes and follicles stored known as follicle reserve at birth is fixed, which cannot be replenished by germ cells in postnatal ovaries, but with age, periodic ovulation, follicle atresia and apoptosis are the main causes of a sharp decline in the number of follicles (Faddy, 2000; Bristol-Gould et al., 2006; Broekmans et al., 2007; Djahanbakhch et al., 2007; Notarianni, 2011; Zhang et al., 2012). Follicle depletion, in turn, causes lower levels of estrogen secretion in the body, eventually leading to menopause (Lawson et al., 2003).

Recently, we report that *Tet2* deficiency accelerates reproductive aging in the adult female mice (Wang et al., 2020). Here we compared the fertility of mice deficient for *Tet1* from young to reproductive old age. We report roles of *Tet1* in maintaining oocyte quality in addition to oocyte number for preserving fertility with age.

## Materials and Methods

### *Tet1* Knockout Mice

*Tet1* knockout (*Tet1^–/–^*) mice were generated from 129 × C57BL/6J mixed genetic background (Zhang et al., 2013). Most of wild-type (WT) and *Tet1* knockout mice were generated from *Tet1* heterozygotes mating. For fertility analysis, *Tet1^–/–^* females were crossed with WT male at young age and vaginal plugs were checked daily. All mice in the study were maintained in a mixed 129 × C57BL/6 background. All mouse experiments were carried out in accordance with the guidelines and regulations and approved by the Institutional Animal Care and Use Committee of Nankai University.

### Genotyping

Postnatal mice at 2 weeks old were genotyped using DNA extracted from their ears. PCR was carried out at 94°C for 2 min, followed by 35 cycles at 94°C for 30 s, 60°C for 30 s, and 72°C for 1 min. DNA fragments were visualized by agarose gel electrophoresis. The genotyping primers were listed as follows.

**Table:** 

*Tet1-C*	CAGTAGTATTTTGCCTGCCTGCAT
*Tet1-R*	TTCCCTAAGGAGTTTACTGCAACG
*Tet1-F*	CATCCTAAATAACCCAACCACCAA

### H&E of Ovary and Follicle Count

Ovaries were collected from different group mice (*n* > 10 mice for young and middle-age group and *n* = 7–8 mice for old group) and fixed by immersion in 4% paraformaldehyde (PFA) overnight at 4°C, and tissues were embedded with paraffin wax, based on previous methods (Liu et al., 2013). The follicles were categorized into primordial and primary, secondary and antral, accordingly (Myers et al., 2004). Follicles were classified as primordial and primary if they contained an oocyte surrounded by a single layer of squamous, or, cuboidal granulosa cells. Secondary follicles were identified as having more than one layer of granulosa cells with no visible antrum. Antral follicles possessed one or two small areas of follicular fluid (antrum) or a single large antral space.

### Oocyte Collection

For collection of oocytes for RNA-seq, female mice from the different groups were superovulated by intraperitoneal injection of 5 I U pregnant mare’s serum gonadotrophin (PMSG), followed 46-48 h later by 5 I U human chorionic gonadotrophin (hCG), to obtain MII oocytes. Granulosa cells attached to oocytes were removed in M2, or HKSOM supplemented with 0.03% hyaluronidase by gently pipetting.

### Quantitative Real-Time PCR

RNA was isolated from ovaries using RNeasy mini kit (Qiagen), and subject to cDNA synthesis using Moloney Murine Leukemia Virus Reverse Transcriptase (Invitrogen). PCR reactions were set up in duplicates using the FastStart Universal SYBR Green Master (4913914001, Roche) and run on the Mastercycler^®^ RealPlex2 real time PCR detection system (Eppendorf). The final PCR reaction volume in 20 μl contained 10 μl SYBR Green PCR Master Mix, 1 μl cDNA template, 2 μl primer mixture and 7 μl water. Thermal cycling was carried out with a 10 min denaturation step at 95°C, followed by two-step cycles, 15 s at 95°C and 1 min at 60°C. Each sample was analyzed using GAPDH as the internal control. Primers were designed as follows.

**Table:** 

*Tet1-Foward*	CCTCACAGGCACAGGTTACA
*Tet1-Reverse Gapdh-Foward Gapdh-Reverse*	ATTTGGGGCCATTTACTGGT TCAACAGCAACTCCCACTCTTCCA ACCACCCTGTTGCTGTAGCCGTAT

### Immunofluorescence of Spreads, Sections, and Oocytes

For paraffin sections, after deparaffinizing, rehydrating and washing in PBS (pH 7.2–7.4), the sections were subjected to high pressure antigen recovery sequentially in citrate buffer (pH 6.0) for 3 min, incubated with blocking solution (5% goat serum and 0.1% BSA in PBS) for 2 h at room temperature, and then incubated with the diluted primary antibodies [anti-5 hmC (39769, Active Motif), anti-Tet1 (MABE1144, Millipore), anti-Oct4 (SC-5279, Santa Cruz)] overnight at 4°C. After washing with PBS, sections were incubated with appropriate secondary antibodies (Alexa Fluor^®^ FITC, 488, or 594). The sections were then stained with 1 μg/ml DAPI for 10 min to reveal nuclei, washed with PBS, and mounted in Vectashield (H-1000, Vector Laboratories, Burlingame, CA, United States). Relative integrated fluorescence intensity for 5 hmC was estimated by Image J software. The threshold was defined using non-specific background fluorescence.

### Single-Cell Isolation and Lysis

MII oocytes were collected from young WT (*n* = 19), *Tet1^–/–^* (*n* = 15) or from old WT (*n* = 19), *Tet1^–/–^* (*n* = 14) mice in two biologically repeated experiments. Single MII oocyte was resuspended in PBS with 0.1% BSA (A3311-10g, Sigma), picked up in 1 μL 0.1% BSA using a micropipette with an epT.I.P.S. pipette tip (0030000838, Eppendorf) under a dissecting microscope, and transferred to the bottom of a 200-μL PCR tube (8-strip, nuclease-free, thin-walled PCR tubes with caps, PCR-0208-C, Axygen) containing oligo (dT) primer, Triton X-100, and Recombinant RNase Inhibitor (RRI, 2313A, Takara). Samples were frozen in liquid nitrogen and stored or used immediately.

### Reverse Transcription

Frozen or fresh samples were melted on ice and 1 μL deoxy-ribonucleoside triphosphate (R0191, Thermo Scientific) was added into the tubes, vortexed gently, and incubated at 37°C for 3 min. The cDNAs were then synthesized using SuperScript^TM^ II Reverse Transcriptase (18064071, Thermo Scientific) by slightly modified Smart-seq2 methods (Picelli et al., 2014), followed by 15 cycles of PCR using KAPA HotStart ReadyMix (KK2602, KAPA Biosystems) and then purified using Agencount AMPure XP beads (A63881, Beckman).

### Library Construction and Sequencing

RNA-seq libraries were prepared using Smart-seq2 methods as previously described (Picelli et al., 2014). Briefly, the libraries were prepared by using TruePrep DNA Library Prep Kit V2 for Illumina^®^ (TD503-02, Vazyme Biotech co.,ltd.) according to the manual instruction. Samples were barcoded during library preparation and multiplex sequenced, with a 150-bp pair-end sequencing strategy on a HiSeq 10x (Illumina).

### Single-Cell RNA-seq Data Analysis

Raw reads were processed using trim-galore and clean reads were mapped to mm 10 from UCSC genome^1^ by Hisat2 (v2.1.0) with default parameters (Kim et al., 2013, 2015). Uniquely mapped reads annotated in Gencode vM17 were calculated by Featurecounts (Liao et al., 2014), with default parameters (featureCounts −T 30 −O −Q 30 −p −a gencode.vM17.annotation.gtf.gz -o v17.txt ^∗^.bam). Sum factor normalization was applied with deconvolution of size factors within different batch samples using SCnorm (Bacher et al., 2017). Raw counts were normalized by library size via counts of exon model per million mapped reads (CPM). Gene expression was counted in individual oocyte when the transcript number was >1. Dynamic changes of differentially expressed genes (DEGs) between different groups were analyzed using Deseq2 (Love et al., 2014). DEGs were defined only if *p* value was <0.05, and a fold change was >1.5. Young *Tet1^–/–^* oocytes had 2,013 downregulated and 1,557 upregulated genes, compared with those of young WT oocytes. There were 1,448 downregulated and 1,696 upregulated genes in old *Tet1*^–/–^ oocytes compared with those of old WT oocytes.

### Functional Annotation

Gene ontology (GO) terms were collected from MGI (Bult et al., 2019) and kyoto encyclopedia of genes and genomes (KEGG) pathways from KEGG pathway database (Wixon and Kell, 2000). Enrichment results were obtained using clusterprofiler (Yu et al., 2012) and KOBAS (Xie et al., 2011).

### Analysis of Transposable Elements

Reads were mapped to mm 10 from UCSC genome see text footnote 1 by Hisat2 (v2.1.0) with default parameters as above. For estimation of retroelement expression, “repeatMasker” track, was downloaded from UCSC genome browser in GTF, based on the genomic coordination in GTF files. Annotation file of transposable elements was extracted and made as a SAF file, which is uploaded in https://github.com/LianaLiu/single. Reads were counted using featurecounts and only the unique mapped reads with completely overlapping retrotransposon coordinates were counted. Class and family of transposable elements are also provided in the supplementary datasheet Supplementary Table 1. Annotation of full-length retrotransposon elements was constructed based on methods described previously (Kaul et al., 2019).

Full length of L1 annotation file was also analyzed according to the previous method (Deininger et al., 2017; Kaul et al., 2019), with slight modifications, and 6,000 bp of L1 sequence were assumed to be the full-length elements. We utilized our paired-end RNA-seq reads aligned by Hisat2 and Samtools to create a sorted bam file and annotated based on our data.

### Hormone Assays

Serum anti-Mullerian hormone (AMH) levels were assayed using ELISA kit (CK-E90200, Hangzhou EastBiopharm CO., LTD.). Quality control serum, sterilized distilled water, and five series diluted standard samples for a standard curve were tested for each serum sample. The intra- and inter-assay coefficients of variability for AMH were below 8 and 12%.

### Statistical Analysis

Data were analyzed by student’s *t*-test, χ2 test or fisher’s exact test for paired comparison, or by ANOVA and means compared by Fisher’s protected least-significant difference (PLSD) for multiple comparisons using the StatView from SAS Institute and the graphs made by GraphPad Prism 7 (GraphPad Software, San Diego, CA, United States). Kruskal-Wallis test was used to compare multiple groups using ggpubr R package. Linear regression analysis was done using GraphPad Prism 7. The data between groups were considered significant when *P* < 0.05 (^∗^), 0.01(^∗∗^) or 0.001(^∗∗∗^).

## Results

### *Tet1* Deficiency Reduces Follicle Reserve and Causes Premature Ovarian Failure and Infertility

To study whether Tet1 regulates fertility with age, we employed *Tet1* deficient mice generated using homologous recombination by deleting exons 11-13 (Zhang et al., 2013). Homozygous *Tet1* knockout mice (*Tet1*^–/–^) were produced by crossing heterozygous mice (Supplementary Figure 1A). However, *Tet1* deficiency resulted in death of a small proportion of embryos (Supplementary Figure 1B), consistent with previous study (Yamaguchi et al., 2012). The *Tet1* knockout was evaluated based on genotyping, as well as the minimal mRNA expression level of *Tet1* determined by qPCR (Supplementary Figure 1C). Also, Tet1 protein was absent in *Tet1*^–/–^ blastocysts by immunofluorescence microscopy (Supplementary Figure 1D), further validating the successful knockout of the *Tet1* gene. Representative primordial oocytes were shown by enclosed granulosa cells (Supplementary Figure 1E). The 5 hmC levels of primordial oocytes from *Tet1*^–/–^ mice as shown by immunofluorescence were decreased compared to WT oocytes (Supplementary Figure 1F).

**FIGURE 1:**
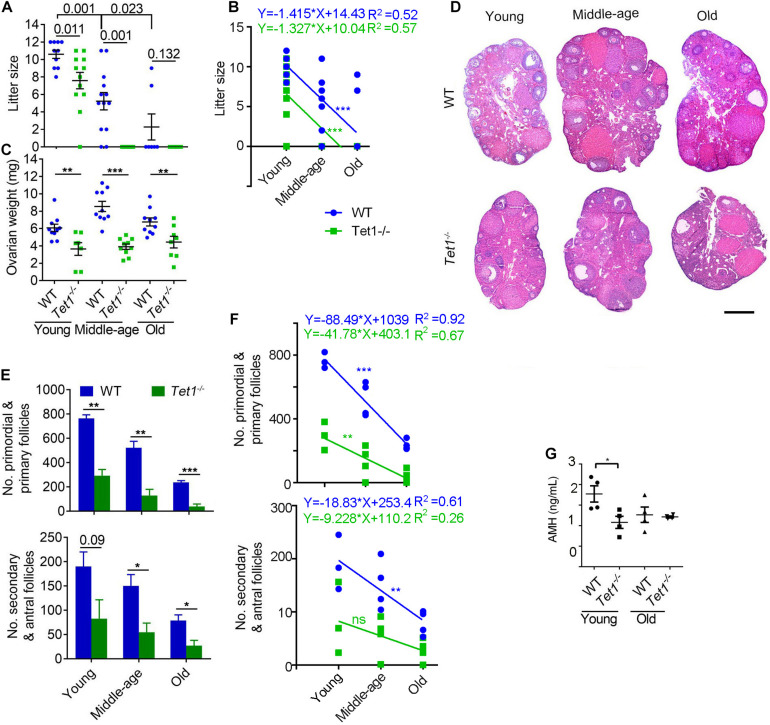
*Tet1* knockout causes subfertility and premature ovarian failure. **(A)** Average litter size of wild-type (WT) and *Tet1* knockout (*Tet1*^–/–^) female mice at three different reproductive age (young, 2–4 months; middle-age, 7–9 months; old, 10–12 months). *n* > 10 mice for young and middle-age group and *n* = 7–8 mice for old group. **(B)** Linear regression analysis of the age effects on litter size between WT and *Tet1*^–/–^ mice. *n* > 10 mice for young and middle-age group and *n* = 7–8 mice for old group. **(C)** Average weight per ovary from WT and *Tet1*^–/–^ mice. *n* > 7. **(D)** Representative images of hematoxylin and eosin (H&E) staining of ovarian sections from young, middle-age and old groups. Scale bar, 500 μm. **(E)** Number of primordial and primary follicles, or secondary and antral follicles from WT and *Tet1^–/–^* mice. *n* = 4-6. **(F)** Linear regression analysis of the age effects on the number of follicles between WT and *Tet1*^–/–^ mice. *n* = 3-4. ns, no significant difference. **(G)** AMH levels in serum of WT and *Tet1^–/–^* mice by ELISA assay (*n* = 4). The bars show mean ± SEM. *P < 0.05; **P < 0.01; ***P < 0.001. The *P* value is labeled if *P* > 0.05 **(A,E)**.

Wild-type (WT) and *Tet1*^–/–^ female mice at the reproductive age of 2–4 months (young), 7–9 months (middle-age) and 10–12 months (old), were mated with young WT males, and the successfully mated females judged by the presence of mating plug next morning were allowed to deliver the pups to determine the fertility. WT mice exhibited reduced fertility with age as shown by smaller litter size, as expected for natural reproductive aging in females. Comparatively, *Tet1*^–/–^ female mice manifested declined fertility and accelerated reproductive failure with age, as they produced smaller litter size than that of age-matched WT mice (Figure 1A). By linear regression analysis, WT and *Tet1*^–/–^ mice with age displayed similar negative slope in the production of the offspring (Figure 1B). While WT females at middle-age produced an average of six pups, *Tet1*^–/–^ deficient age-matched mice or at an older age failed to give birth to a pup (Figure 1A). *Tet1*^–/–^ female mice were infertile at middle-age. This phenomenon is comparable to premature ovarian failure. Consistently, ovaries of *Tet1*^–/–^ females at three age groups were lighter than those of age-matched WT females from young to old age (Figure 1C). By examining the ovarian serial sections by histology, the number of primordial and primary follicles as well as secondary and mature antral follicles was dramatically reduced in *Tet1*^–/–^ females from the three age groups, compared to WT mice served as control (Figures 1D,E). Slope in the declined number of follicles in WT mice with age appeared to be much higher than did *Tet1*^–/–^ mice, and the decrease of secondary and antral follicles in *Tet1*^–/–^ mice with age actually was not significant (Figure 1F). This likely was because the follicle reserve was already dramatically lower in *Tet1*^–/–^ than in WT mice at young age. Consistently, lower AMH levels indicative of ovarian reserve were found in serum of *Tet1*^–/–^ than in WT mice at young age (Figure 1G). Therefore, *Tet1* deficiency decreases fecundity particularly with age. The decreased fecundity is largely caused by prominent reduction of follicle reserve and development, and also probably by reduced oocyte quality.

### Transcriptome Analysis of *Tet1*-Deficient Oocytes in Comparison With WT Oocytes

**FIGURE 2:**
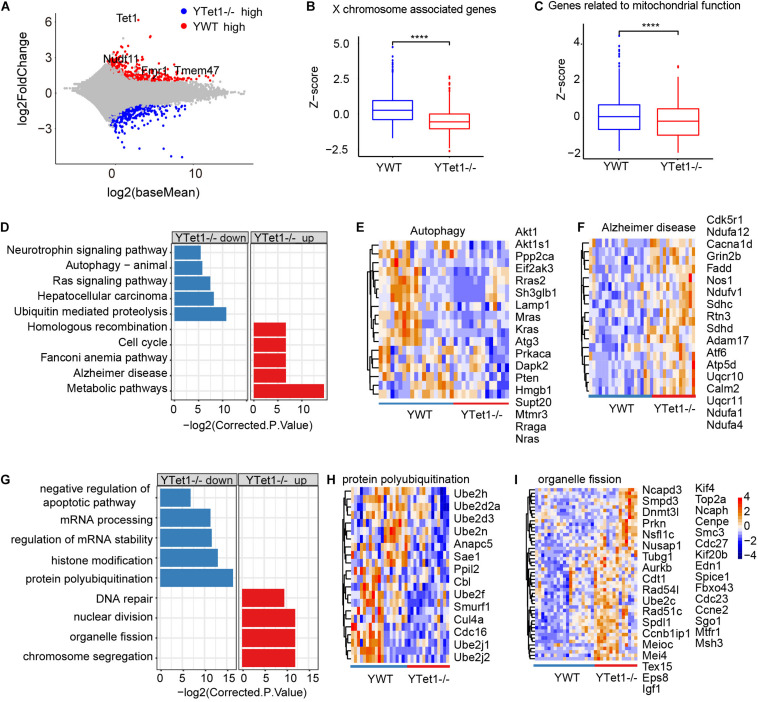
Differential gene expression of *Tet1^–/–^* and WT oocytes. **(A)** Scatter-plots showing gene expression profile between young *Tet1^–/–^* (*n* = 15) and WT (*n* = 19) oocytes from two biological repeats. **(B)** Boxplot showing gene expression on chromosome X and downregulated in young *Tet1^–/–^* oocytes compared with young WT oocytes. ****P < 0.0001. **(C)** Boxplot showing expression of genes on mitochondrial function and Downregulated in young Tet1^–/–^ oocytes. **(D)** KEGG enrichment results showing genes downregulated or upregulated after *Tet1* deficiency. *X*-axis represents the corrected *p* value (FDR) using Benjamini and Hochberg. **(E)** Pheatmap showing genes enriched in autophagy signaling pathway down regulated in *Tet1* deficient oocytes. **(F)** Pheatmap showing genes enriched in Alzheimer’s disease upregulated in *Tet1* deficient oocytes. **(G)** GO enrichment results showing genes downregulated or upregulated after *Tet1* deficiency. *X*-axis represents the corrected *p* value (FDR) using Benjamini and Hochberg. **(H)** Pheatmap showing genes enriched in protein polyubiquitination in *Tet1* deficient oocytes. **(I)** Pheatmap showing genes enriched in organelle fission upregulated in *Tet1* deficient oocytes.

To examine potential molecular changes in the oocytes, we performed single-cell RNA-seq detecting an average number of genes more than 15,000 (Supplementary Figure 2A) to analyze the oocytes of *Tet1*^–^*^/^*^–^ (*n* = 15) and WT (*n* = 19) collected from young mice in two biological repeated experiments. *Tet1*^–^*^/^*^–^ oocytes showed 2,013 downregulated (*p* value < 0.05, and a fold change <−1.5) and 1,557 upregulated genes (*p* value < 0.05, and a fold change >1.5), compared with WT oocytes (Figure 2A). Significantly downregulated genes in *Tet1^–/–^* oocytes included *Fmr1* and *Tet1*, supporting an effective deletion of *Tet1*. Also, we analyzed differential gene expression among chromosomes (Supplementary Figure 2A). Notably, gene expression on chromosome X (Figure 2B) and in mitochondrial functions (Figure 2C) was significantly decreased in *Tet1^–/–^* oocytes compared with WT oocytes. About 39 genes located on X-chromosomes such as *Nudf11*, *Tmem47*, and *Fmr1* were down-regulated in *Tet1^–/–^* oocytes. Due to the important roles of *Fmr1*, we used metascape to analyzed the potential protein-protein interaction enrichment analysis and found Fmr1 might interact with *Hnrnpu* and *Pabpc1* (Supplementary Figure 2B). Moreover, the upregulated genes by *Tet1* deficiency were enriched in homologous recombination, cell cycle, fanconi anemia pathway, Alzheimer’s disease, and metabolic pathways, while downregulated genes enriched in Ras signaling pathway, neurotrophin signaling pathway, which can cause premature ovarian failure (Dorfman et al., 2014), and notably autophagy and ubiquitin mediated proteolysis (Figures 2D–F). By GO enrichment analysis, downregulated genes after *Tet1* deficiency were enriched in mRNA processing, regulation of mRNA stability, histone modification, negative regulation of apoptotic pathway such as *Uri1*, *Rtkn2*, *Nr4a2, Bcl2l2, Akt1* and *Psme3*, and also protein polyubiquitination (Figures 2G,H). Interestingly, genes for DNA repair, nuclear division, chromosome segregation and organelle fission were upregulated in *Tet1*-deficient oocytes (Figures 2G,I). Increased apoptosis has been implicated in reduction of germ cell numbers (Yamaguchi et al., 2012). Together, these results suggest that elevated organelle fission and reduced protein polyubiquitination and autophagy might be implicated in reduced oocyte quality resulting from loss of *Tet1*.

Further, we compared our single cell RNA-seq data on oocytes with published data on PGCs (Yamaguchi et al., 2012) and found that 63 genes including *Rnf8*, *Tet3*, *Fmr1nb*, and *Xlr5a/b/c* were commonly downregulated in both *Tet1*-deficient oocytes and PGCs (Supplementary Figures 3A,B). We also compared our data using oocytes with the differentially expressed genes (DEG) associated with differential methylated regions (DMR) from published data using PGCs (Yamaguchi et al., 2012). About 20 genes, including *Tet1*, *Sycp3*, *Dazl*, and *Sirt6*, exhibited reduced expression in association with increased methylation after *Tet1* deficiency (Supplementary Figure 3C). The analysis shows that Tet1 also plays a role in maturing oocytes in addition to its function in PGCs and meiosis.

### Transcriptome Analysis of Old *Tet1*-Deficient Oocytes

**FIGURE 3:**
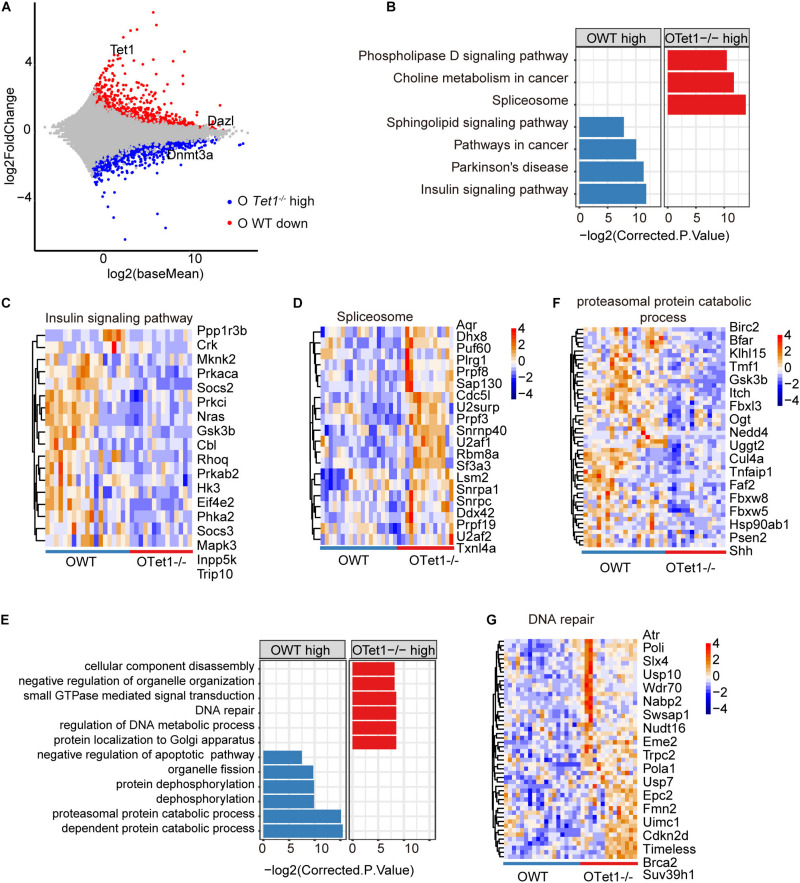
Differential gene expression of old *Tet1^–/–^* and WT oocytes. **(A)** Scatter-plots showing gene expression profile between old *Tet1^–/–^* (*n* = 14) and WT (*n* = 19) oocytes from two biological repeats. **(B)** KEGG enrichment results showing genes downregulated or upregulated after *Tet1* deficiency. *X*-axis represents the corrected *p* value (FDR) using Benjamini and Hochberg. **(C)** Pheatmap showing genes enriched in insulin signaling pathway in *Tet1* deficient oocytes. **(D)** Pheatmap showing genes enriched in spliceosome in *Tet1* deficient oocytes. **(E)** GO enrichment results showing genes downregulated or upregulated after *Tet1* deficiency. *X*-axis represents the corrected *p* value (FDR) using Benjamini and Hochberg. **(F)** Pheatmap showing genes enriched in proteasomal protein catabolic process in *Tet1* deficient oocytes. **(G)** Pheatmap showing genes enriched in DNA repair in *Tet1* deficient oocytes.

We further analyzed differential gene expression between old WT (*n* = 19) and *Tet1^–/–^* oocytes (*n* = 14) in two biological repeats. *Tet1*, as expected, and *Dazl* were downregulated in old *Tet1^–/–^* oocytes compared with old WT oocytes, while *Dnmt3a* was highly expressed in old *Tet1^–/–^* oocytes (Figure 3A). Downregulated genes in old *Tet1^–/–^* oocytes were enriched in insulin signaling pathway, Parkinson’s disease, pathways in cancer and sphingolipid signaling pathway, and genes upregulated in old *Tet1^–/–^* oocytes enriched in spliceosome, choline metabolism in cancer, and phospholipase D signaling pathway (Figure 3B). The expression of genes enriched in insulin signaling pathway (Figure 3C), were decreased and genes enriched in spliceosome (Figure 3D) were increased in *Tet1^–/–^* oocytes. By GO enrichment analysis, genes upregulated in old *Tet1^–/–^* oocytes were enriched in cellular component disassembly, negative regulation of organelle organization, small GTPase mediated signal transduction, DNA repair, regulation of DNA metabolic process and protein localization to Golgi apparatus, whereas genes downregulated enriched in organelle fission, protein dephosphorylation, proteasomal protein catabolic process and negative regulation of apoptotic pathway including genes including *Akt1*, *Bcl2*, *Nr4a2*, etc (Figure 3E). Expression of genes enriched in proteasomal protein catabolic process were decreased (Figure 3F), and those in DNA repair were increased (Figure 3G). Implication of increased DNA repair is not known, but possibly suggests increased DNA damage stress due to loss of *Tet1.*

**FIGURE 4:**
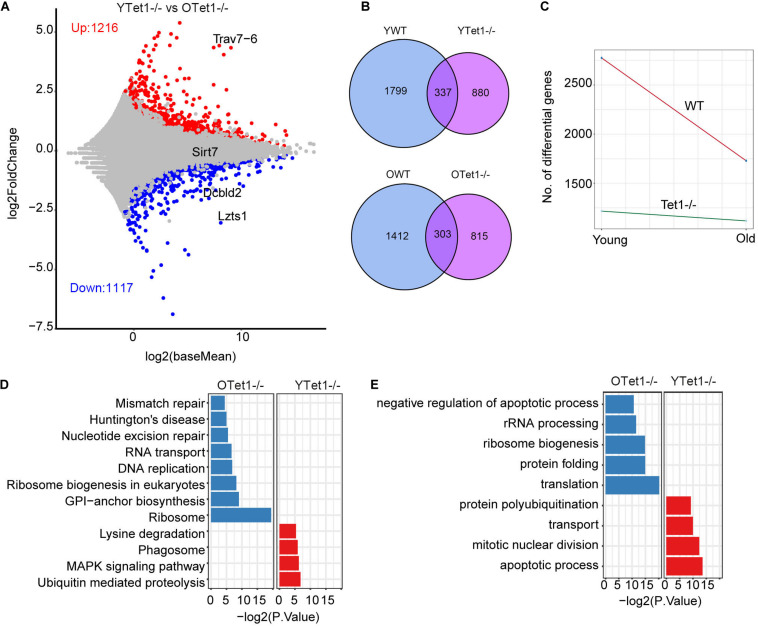
Differential gene expression between young and old *Tet1*^–/–^ oocytes. **(A)** Scatter plot displaying differential gene expression between young and old *Tet1*^–/–^ oocytes. **(B)** Venn plot showing the upregulated and downregulated genes between young and old WT oocytes, and young and old *Tet1*^–/–^ oocytes. **(C)** Slope plot showing the change between WT and *Tet1*^–/–^ oocytes. **(D)** KEGG enrichment results of differential genes between young and old *Tet1*^–/–^ oocytes. *X*-axis represents the *p*-value. **(E)** GO enrichment results of differential genes between young and old *Tet1*^–/–^ oocytes. *X*-axis represents the *p*-value.

Additionally, we analyzed the differential gene expression between young and old *Tet1*^–/–^ oocytes to explore the aging effect on *Tet1*-deficiency. There were 1,117 upregulated and 1,216 downregulated genes in old *Tet1*^–/–^ mouse oocytes compared with young *Tet1*^–/–^ oocytes (Figure 4A). Comparatively, more genes were upregulated and downregulated in old WT oocytes (Figure 4B). By comparing the differential gene number between WT and *Tet1*^–/–^ oocytes with mouse age, interestingly, aging affects gene expression more in WT than in *Tet1*^–/–^ oocytes (Figure 4C). However, the mismatch repair was increased in old *Tet1*^–/–^ oocytes compared with young *Tet1*^–/–^ oocytes by KEGG enrichment analysis (Figure 4D), while apoptotic process was enriched in young *Tet1*^–/–^ oocytes (Figure 4E). These results show that *Tet1* deficiency may overlap some of the aging effect on gene expression. Alternatively, based on complex analysis above, *Tet1* deficiency induces partial aging effects on oocyte function.

### Increase of *LINE1* Transcripts in *Tet1*^–^*^/^*^–^ Oocytes

**FIGURE 5:**
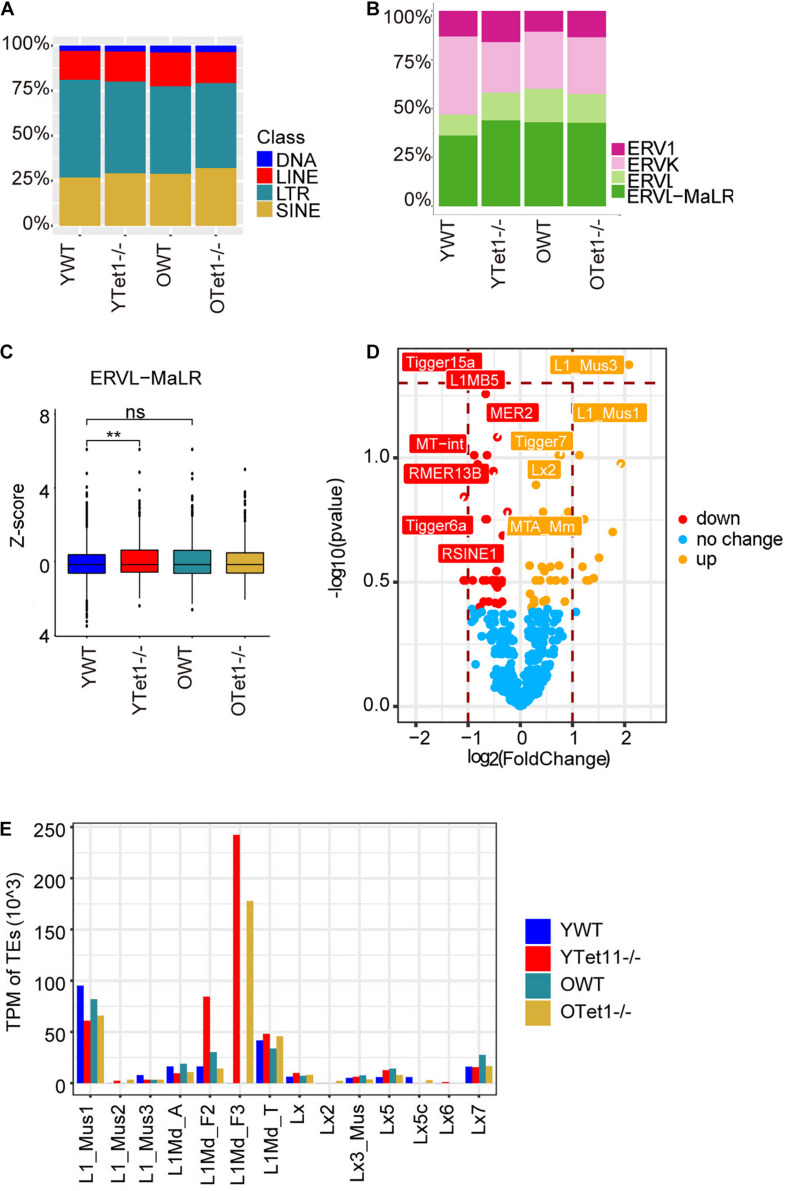
Expression of transposon elements (TEs) of *Tet1^–/–^* and WT oocytes. **(A)** Barplot showing the proportion of TEs in young *Tet1^–/–^*, WT, old *Tet1^–/–^*, and WT oocytes. **(B)** Barplot displaying the proportion of ERVs in young *Tet1^–/–^*, WT, old *Tet1^–/–^*, and WT oocytes. **(C)** *Z*-score showing expression of ERVL-MaLR between young *Tet1^–/–^* and WT oocytes. Data are shown as mean ± SEM. **P < 0.01. **(D)** Volcano showing the differential TEs between young *Tet1^–/–^* and WT oocytes. Orange represented upregulated TEs compared with young WT oocytes. **(E)** Expression of Full-length L1 family in young *Tet1^–/–^* and WT oocytes, and old *Tet1^–/–^* and WT oocytes.

Transposable elements (TEs) constitute approximately 40% of the mouse genome (Bourc’his and Bestor, 2004) and DNA methylation plays roles in repressing TEs (Wu and Zhang, 2010; Cardelli, 2018). Previously, loss of *Tet1* was associated with increased methylation and reduced transcriptional activation of TEs in PGCs (Hill et al., 2018). We analyzed transcripts of TEs in oocytes of young and old mice based on our single cell RNA-seq data. Proportion of *LINE* and *SINE* was slightly higher in *Tet1*^–/–^ oocytes compared with young WT oocytes (Figure 5A). Further analysis of different *ERVs* revealed that the proportion of *ERVL-MaLR* was higher in *Tet1^–/–^* than that in WT oocytes (Figure 5B). Moreover, we used *z*-score to compare the expression of *ERVL-MaLR* belonging to *ERVL-Ma*, and showed that it was increased in *Tet1*^–/–^ oocytes (Figure 5C). To further analyze the difference between young *Tet1^–/–^* and WT oocytes, we performed differential analysis using Deseq2 and found that *L1-mus1* and *L1-mus3* transcripts were increased in young *Tet1*^–/^*^–^* oocytes compared with age-matched WT oocytes (Figure 5D). Based on previous study, only 1/10 L1 elements were full-length with lower coding for retrotranspositionally competent L1 elements (Deininger et al., 2017). We further analyzed the full-length L1 family and found that *L1Md_F3* was notably highly expressed in *Tet1*^–/–^ deficient oocytes, in contrast to WT oocytes (Figure 5E). These results together suggest that *Tet1* deficiency also may alter transcription of TEs in oocytes.

## Discussion

Here, we show that *Tet1* deficiency leads to POF by influencing the quality of oocytes, including aberrant X-chromosome inactivation, and increased expression of L1-mus, as well as the oocyte number and follicle reserve. Previous elegant study demonstrated critical role of *Tet1* in meiosis/PGCs during fetal development (Yamaguchi et al., 2012). *Tet1* deficiency leads to aberrant meiosis, increased methylation on meiosis genes and subfertility in young mice (Yamaguchi et al., 2012). We extended the study by evaluating age effects on the fertility and oocytes in adult mice deficient in *Tet1.* We find that *Tet1* deficient mice at reproductive middle-age already are completely infertile, consistent with POF or primary ovarian insufficiency (POI), which is characterized by the premature depletion of ovarian follicles and infertility at mid-reproductive age (Shah and Nagarajan, 2014; Delcour et al., 2019).

We show that *Tet1* deficiency upregulates genes for Alzheimer’s diseases, but reduces expression of X-chromosome-linked genes, such as *Fmr1*. These defects might be linked in POF. Women with primary ovarian insufficiency (POI) or POF are at increased risk for non-communicable diseases such as, Alzheimer’s disease, cardiovascular disease, and osteoporosis (Harlow and Signorello, 2000; Shah and Nagarajan, 2014). POF has repeatedly been associated to X-chromosome deletions and haploinsufficiency of X-linked genes can be on the basis of POF (Ferreira et al., 2010). X chromosome inactivation could underlie POF linked to cognitive impairment in Alzheimer’s disease (Al-Hinti et al., 2007; Bretherick et al., 2007; Davey, 2013), regardless existing discrepancy on X chromosome in POF. Moreover, fragile X mental retardation type 1 (*FMR1*) gene premutation on the X chromosome have been frequently found in POF or POI (Jin et al., 2012; Shamilova et al., 2013; Bouali et al., 2015; Qin et al., 2015; Mila et al., 2018). FMR1 gene premutation allele’s carrier women have an increased risk for, or, susceptibility to POF (Ferreira et al., 2010; Pu et al., 2014). *FMR1* gene premutation is the first single-gene cause of primary ovarian failure (Fragile X-associated primary ovarian insufficiency) and one of the most common causes of ataxia (fragile X-associated tremor/ataxia syndrome), and multiple additional phenotypes including neuropathy and neuropsychiatric alterations (Mila et al., 2018).

Moreover, loss of *Tet1* increases DNA methylation as shown by increased expression of Dnmt3a and declined 5hmC levels in oocytes. Tet enzyme can regulate DNA demethylation as well as transcription (Wu and Zhang, 2017; Greenberg and Bourc’his, 2019). DNA methylation is one of the best-characterized epigenetic modifications and has been implicated in numerous biological processes, including transposable element silencing, genomic imprinting and X chromosome inactivation (Wu and Zhang, 2010; Dai et al., 2016). Oxidation of 5-methylcytosine by TET dioxygenases can lead to global demethylation (Dai et al., 2016; Wu and Zhang, 2017). About 60–80% of the CpG sites in the mammalian genome are modified by 5 mC (Smith and Meissner, 2013). TET-mediated oxidation has a locus-specific effect (Yamaguchi et al., 2012, 2013b; Dawlaty et al., 2013; Wu and Zhang, 2017). For instance, integrative Genomics Viewer reveals that the binding of Tet1 and 5 hmC levels on *Fmr1* locus in WT is decreased in *Tet1*-knockdown ESCs by analysis of published data GEO datasets (GSE24841) (Williams et al., 2011), likely reducing expression of *Fmr1*. Indeed, blocking DNA methylation by 5-azacytidine (5-azaC or 5-azadC) has achieved a significant reactivation of *FMR1* gene expression in fragile X syndrome cellular models (Bar-Nur et al., 2012). Also, L1 and ERVL-MaLR are aberrantly expressed in *Tet1*-deficient oocytes. *L1* is highly expressed during mouse oocyte development and embryo cleavage stage (Jachowicz et al., 2017). But, elevated L1 expression correlates with fetal oocyte attrition, oocyte aneuploidy and embryonic lethality (Malki et al., 2014).

Additionally, *Tet1* deficiency results in declined ubiquitination and autophagy but increased organelle fission in oocytes, and these defects presumably can be detrimental to clearance of damaged, or, senescent organelles in the cells. Impaired autophagy can lead to POF (Gawriluk et al., 2014; Delcour et al., 2019). *Tet1* deficiency also decreases Ras signaling pathways. Appropriate activation of RAS signaling is crucial for directing normal follicle development (Fan et al., 2008).

Further experiments are required to investigate the underlying molecular mechanisms of how *Tet1* regulates these signaling pathways associated with POF or POI. Also, the question remains as to whether Tet1 has similar function in human fertility and in POF patients. Nevertheless, our findings that Tet1 may involve in autophagy, X-chromosome activation and Alzheimer’s disease provide additional insights into molecular basis of POF.

## Data Availability Statement

RNA-seq data have been deposited in the GEO database under the accession number GSE142163.

## Ethics Statement

The animal study was reviewed and approved by the Institutional Animal Care and Use Committee, Nankai University. Written informed consent was obtained from the owners for the participation of their animals in this study.

## Author Contributions

HW performed the experiments on fertility and histology, and prepared the manuscript. LinlLiu analyzed the RNA-seq data and prepared the manuscript. G_LX provided the materials, advised the project, and revised the manuscript. LinLiu conceived the study, designed experiments, and wrote the manuscript. All authors contributed to the article and approved the submitted version.

## Conflict of Interest

The authors declare that the research was conducted in the absence of any commercial or financial relationships that could be construed as a potential conflict of interest.

## Footnotes

1 http://genome.ucsc.edu/

## Supplementary Material

Supplementary Figure 1 Loss of *Tet1* in female mice reduces 5 hmC levels in oocytes. **(A)** PCR genotyping of *Tet1* mutant mice. Primer sequences are listed in Supplementary Table 2. **(B)** Table summarizing the litter size and Mendelian ratio of *Tet1^–/–^* mice. **(C)** *Tet1* mRNA expression level in ovaries of young mice by qPCR analysis. **(D)** Tet1 protein expression level in E3.5 blastocysts by immunofluorescence. Scale bar, 10 μm. **(E)** Representative images of primordial follicles from young WT mouse ovary. **(F)** Representative images of primordial follicles stained with 5 hmC antibody. Dashed line indicates the primordial oocyte. Scale bar, 10 μm. Bottom panel, Relative 5 hmC level in primordial oocytes from young mice. *n* = 21 oocytes counted for each group. Data represents mean ± SEM. ****P* < 0.001.

Supplementary Figure 2 Distribution of differentially expressed genes across chromosomes. **(A)** Scatter plot illustrating the chromosomal distribution of differential genes Between young WT and *Tet1^–/–^* oocytes. **(B)** Protein-protein interactome network analysis using Metascape (http://metascape.org/gp/index.html#) showing the potential interaction of Fmr1, Hnrnpu and Pabpc1 and with other proteins.

Supplementary Figure 3 Coverage of Smart-seq2 of oocytes and comparison with transcriptome data on PGCs from Yamaguchi et al., 2012. **(A)** Boxplot displaying number of genes sequenced in oocytes. **(B)** Venn plot showing the overlapped, downregulated genes between oocytes based on our data and PGCs from Yamaguchi et al., 2012. **(C)** Venn plot showing the overlapped, downregulated genes between oocytes and PGCss associated with DMRs, based on Yamaguchi et al., 2012.
