# Supplementary material for: Tet1 Deficiency Leads to Premature Ovarian Failure
Source: Front Cell Dev Biol. 2021 Mar 23;9:644135. doi: 10.3389/fcell.2021.644135 (PMC8021788; doi:10.3389/fcell.2021.644135)
Supplement: Supplementary Figure 1 — Loss of Tet1 in female mice reduces 5 hmC levels in oocytes. (A) PCR genotyping of Tet1 mutant mice. Primer sequences are listed in Supplementary Table 2. (B) Table summarizing the litter size and Mendelian ratio of Tet1–/– mice. (C) Tet1 mRNA expression level in ovaries of young mice by qPCR analysis. (D) Tet1 protein expression level in E3.5 blastocysts by immunofluorescence. Scale bar, 10 μm. (E) Representative images of primordial follicles from young WT mouse ovary. (F) Representative images of primordial follicles stained with 5 hmC antibody. Dashed line indicates the primordial oocyte. Scale bar, 10 μm. Bottom panel, Relative 5 hmC level in primordial oocytes from young mice. n = 21 oocytes counted for each group. Data represents mean ± SEM. ***P < 0.001. [file Data_Sheet_2.docx]

***Supplementary Figures***

***Tet1* deficiency leads to premature ovarian failure**

Linlin Liu, Huasong Wang, Guo_Liang Xu, and Lin Liu

**
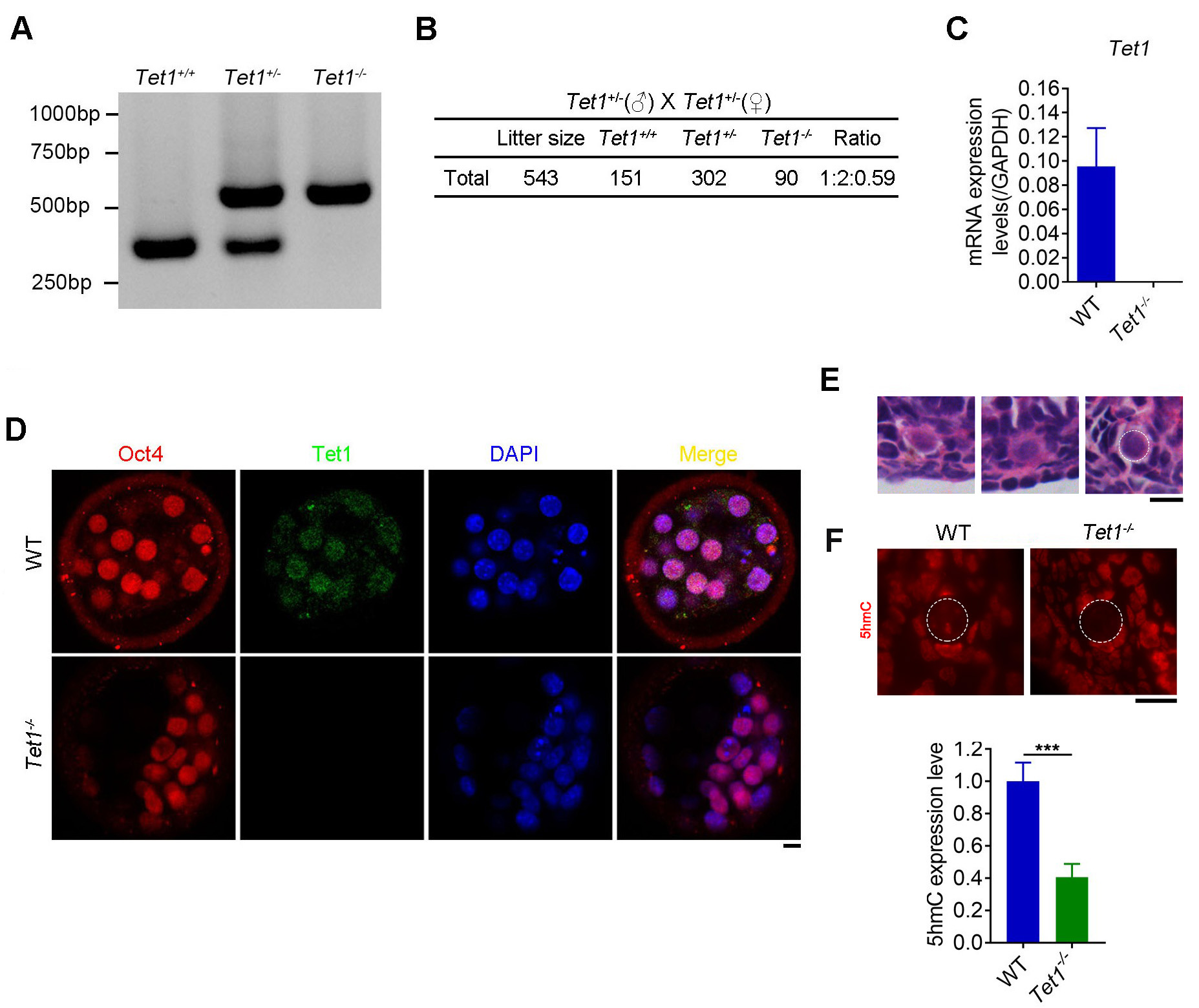
**

**Figure S1. Loss of *Tet1* in female mice reduces 5hmC levels in oocytes.**

(A) PCR genotyping of *Tet1* mutant mice. Primer sequences are listed in Table S2.

(B) Table summarizing the litter size and Mendelian ratio of *Tet1^-/-^* mice.

(C) *Tet1* mRNA expression level in ovaries of young mice by qPCR analysis.

(D) Tet1 protein expression level in E3.5 blastocysts by immunofluorescence. Scale bar, 10 μm.

(E) Representative images of primordial follicles from young WT mouse ovary.

(F) Representative images of primordial follicles stained with 5hmC antibody. Dashed line indicates the primordial oocyte. Scale bar, 10 μm. Bottom panel, Relative 5hmC level in primordial oocytes from young mice. n=21 oocytes counted for each group. Data represents mean ± SEM. ***P<0.001.


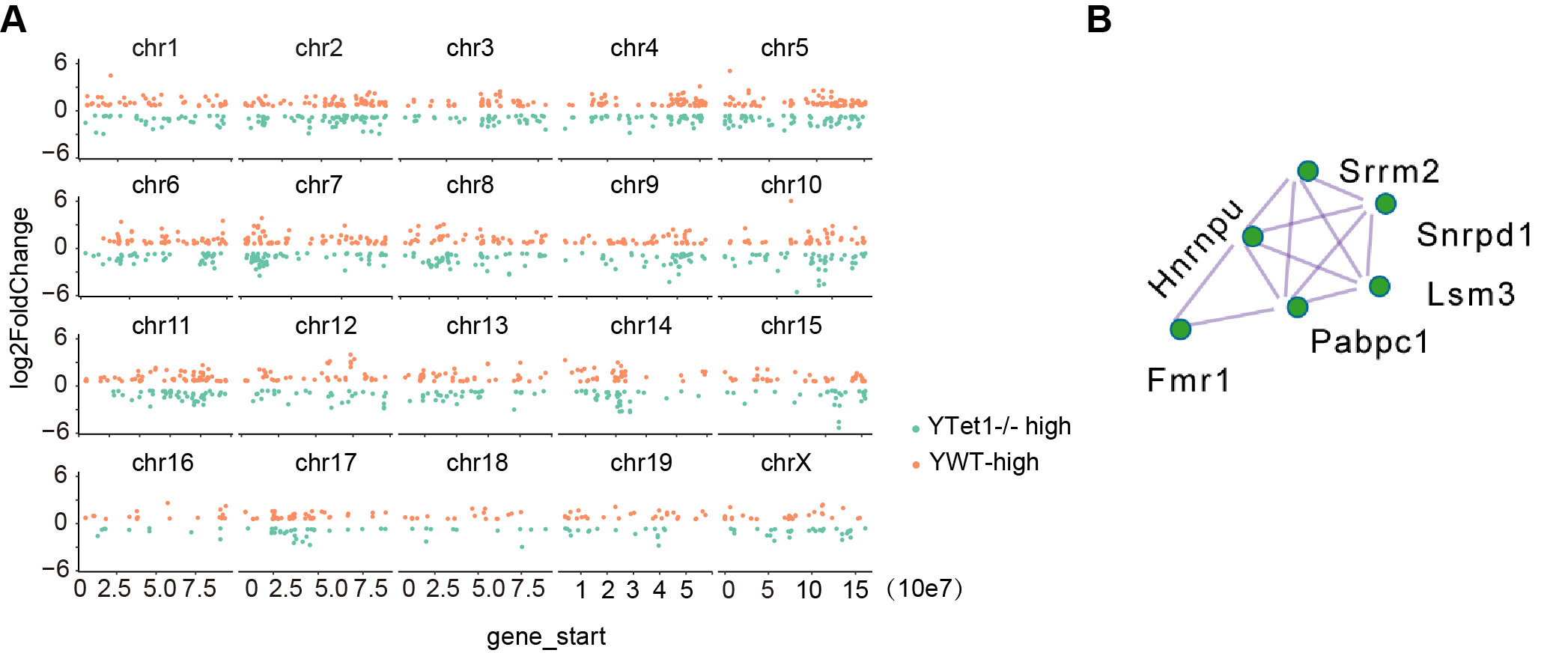


**Figure S2. Distribution of differentially expressed genes across chromosomes.**

1. Scatter plot illustrating the chromosomal distribution of differential genes Between young WT and *Tet1^-/-^* oocytes.
2. Protein-protein interactome network analysis using Metascape ([http://metascape.org/gp/index.html#](http://metascape.org/gp/index.html)) showing the potential interaction of Fmr1, Hnrnpu and Pabpc1 and with other proteins.

**
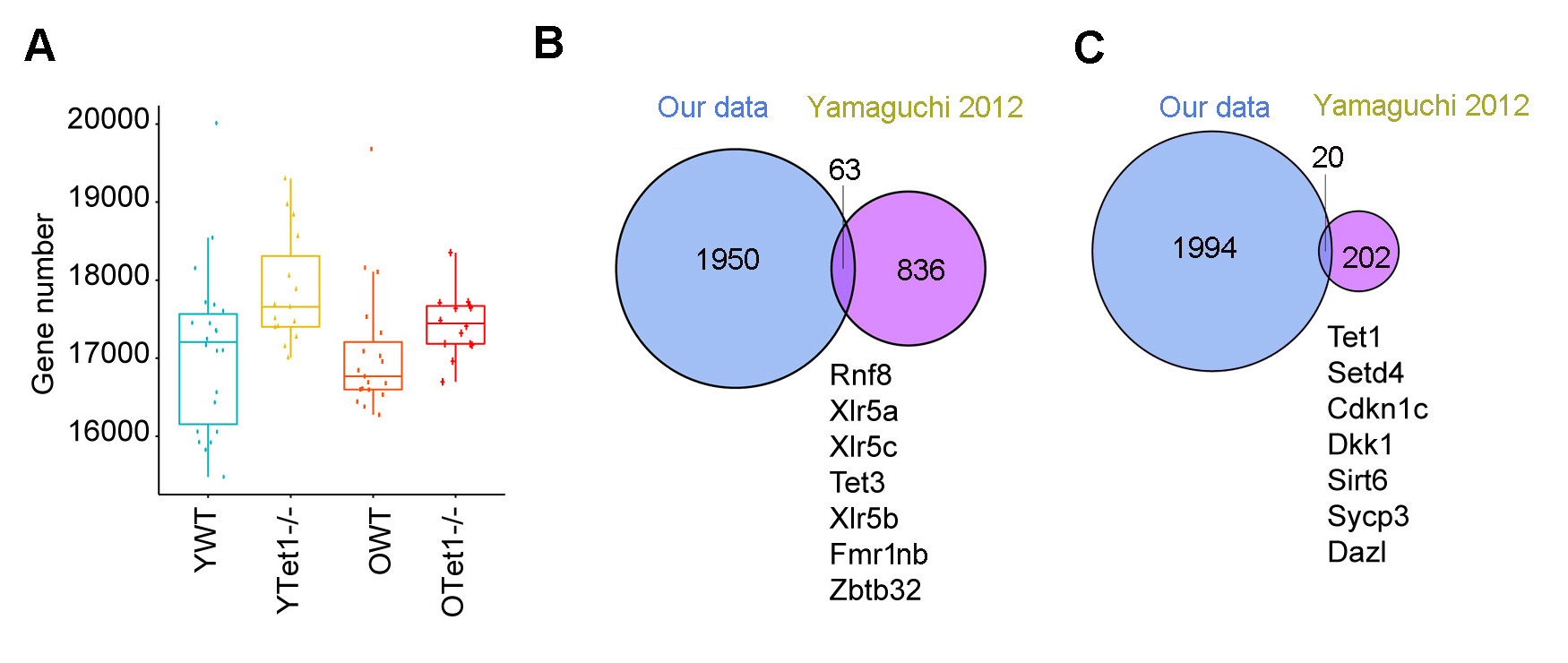
**

**Figure S3. Coverage of Smart-seq2 of oocytes and comparison with transcriptome data on PGCs from Yamaguchi et al. 2012.**

(A) Boxplot displaying number of genes sequenced in oocytes.

(B) Venn plot showing the overlapped, downregulated genes between oocytes based on our data and PGCs from Yamaguchi et al. 2012.

(C) Venn plot showing the overlapped, downregulated genes between oocytes and PGCss associated with DMRs, based on Yamaguchi et al. 2012.
